# Oral Infection by Mucosal and Cutaneous Human Papillomaviruses in the Men Who Have Sex with Men from the OHMAR Study

**DOI:** 10.3390/v12080899

**Published:** 2020-08-17

**Authors:** Tarik Gheit, Francesca Rollo, Rosario N Brancaccio, Alexis Robitaille, Luisa Galati, Massimo Giuliani, Alessandra Latini, Barbara Pichi, Maria Benevolo, Cyrille Cuenin, Sandrine McKay-Chopin, Raul Pellini, Antonio Cristaudo, Aldo Morrone, Massimo Tommasino, Maria Gabriella Donà

**Affiliations:** 1 International Agency for Research on Cancer, 150 Cours Albert Thomas, CEDEX 08, 69372 Lyon, France; gheitt@iarc.fr (T.G.); brancaccior@students.iarc.fr (R.N.B.); robitaillea@students.iarc.fr (A.R.); galatil@students.iarc.fr (L.G.); cueninc@iarc.fr (C.C.); chopins@iarc.fr (S.M.-C.); tommasinom@iarc.fr (M.T.); 2 Pathology Department, Regina Elena National Cancer Institute IRCCS, Via Elio Chianesi 53, 00144 Rome, Italy; francesca.rollo@ifo.gov.it (F.R.); maria.benevolo@ifo.gov.it (M.B.); 3 STI/HIV Unit, San Gallicano Dermatological Institute IRCCS, Via Elio Chianesi 53, 00144 Rome, Italy; massimo.giuliani@ifo.gov.it (M.G.); alessandra.latini@ifo.gov.it (A.L.); antonio.cristaudo@ifo.gov.it (A.C.); 4 Otolorayngology Head&Neck Surgery Department, Regina Elena National Cancer Institute IRCCS, Via Elio Chianesi 53, 00144 Rome, Italy; barbara.pichi@ifo.gov.it (B.P.); raul.pellini@ifo.gov.it (R.P.); 5 Scientific Direction, San Gallicano Dermatologic Institute IRCCS, Via Elio Chianesi 53, 00144 Rome, Italy; aldo.morrone@ifo.gov.it

**Keywords:** Human Papillomavirus, HPV, alpha, beta, gamma, cutaneous, mucosal, oral rinse and gargle, HIV, men who have sex with men, MSM, next generation sequencing, NGS

## Abstract

Both mucosal and cutaneous Human Papillomaviruses (HPVs) can be detected in the oral cavity, but investigations regarding the epidemiology of cutaneous HPVs at this site are scarce. We assessed mucosal (alpha) and cutaneous (beta and gamma) HPV infection in oral samples of HIV-infected and uninfected men who have sex with men (MSM). Oral rinse-and-gargles were collected from 310 MSM. Alpha HPVs were detected using the Linear Array, whereas beta and gamma HPVs were detected using multiplex PCR and Luminex technology. An amplicon-based next-generation sequencing (NGS) protocol was applied to a subset of samples collected from 30 HIV-uninfected and 30 HIV-infected MSM. Beta HPVs were significantly more common than alpha types (53.8% vs. 23.9% for HIV-infected subjects, *p* < 0.0001; 50.3% vs. 17.1% for HIV-uninfected subjects, *p* < 0.0001). Gamma HPVs were also frequently detected (30.8% and 25.9% in HIV-infected and uninfected MSM, respectively). NGS produced 2,620,725 reads representative of 146 known HPVs (16 alpha-PVs, 53 beta-PVs, 76 gamma-PVs, one unclassified) and eight putative new HPVs, taxonomically assigned to the beta genus. The oral cavity contains a wide spectrum of HPVs, with beta types representing the predominant genus. The prevalence of beta and gamma HPVs is high even in immunorestored HIV-infected individuals. NGS confirmed the abundance of cutaneous HPVs and identified some putative novel beta HPVs. This study confirms that cutaneous HPVs are frequently present at mucosal sites and highlights that their pathological role deserves further investigation since it may not be limited to skin lesions.

## 1. Introduction

Recent studies indicated that cutaneous Human Papillomaviruses (HPVs), which mainly belong to the beta and gamma genera, can be found in mucosal epithelia. Indeed, they can be detected in the anal canal [1,2,3], nasal mucosa [4], and oral cavity [5,6,7]. Beta HPVs were observed in around 12% of oral samples in a population-based study [5] and 20–40% of mostly heterosexual men [6], as well as 20% of mid-adult women [7]. Beta HPVs have been detected across multiple cutaneous and mucosal sites of the same subjects, although the concordance across the different sites was generally low, especially between the oral and ano-genital ones [8]. Oral infection by gamma HPVs appears to be less common, with a prevalence ranging from 3% to 11% [5,7]. A recent study confirmed the presence of these genotypes in oral swabs using broad-spectrum primer systems [9]. Although frequency of HPV oral infection in this study was low, especially compared to other mucosal sites, and alpha HPVs were the most frequent types detected in oral swabs, beta and gamma HPVs were also found. Notably, a large prospective study estimated an incidence rate for cutaneous HPV oral infection of 4.17 × 1000 person-months, which is almost 10 times higher than that of mucosal HPV oral infection [10]. Nonetheless, the same investigation showed a similar median time to clearance for both cutaneous and mucosal HPVs, i.e., 6 months.

The frequent detection of cutaneous HPVs in oral samples certainly leads to questions regarding their possible pathologic role at head and neck site. Although their pathologic role at mucosal epithelia still remains unknown, it is worth noting that cutaneous HPVs were found in a minor fraction of head and neck papillomas [11,12]. Interestingly, a longitudinal study provided evidence that oral positivity for some beta or gamma HPVs is associated with the development of head and neck cancer [13], although a direct role of cutaneous HPVs in this neoplasia remains to be proven. Certain beta HPVs play a role in the development of cutaneous squamous cell carcinoma and are classified as “possibly carcinogenic” in epidermodysplasia verruciformis patients [14].

Epidemiologic determinants of oral infection by mucosal and cutaneous HPVs in the general population seem to differ [5]. Sexual behavior is clearly a determinant of oral infection by alpha HPVs [5,15,16,17]. Regarding beta HPV types, fingers may represent a source of viral transmission to the oral cavity [7]. There are also indications for sexual transmission routes for beta and gamma HPVs [7], despite the fact that other studies did not confirm this association [5,6,7].

It is well established that men who have sex with men (MSM) [18,19], especially those with HIV-1 infection [20], harbor an increased risk of acquiring an HPV infection, also of the oral mucosa [21,22]. Additional studies to investigate the spectrum of HPVs present at this site are required to better characterize oral HPV infection and cutaneous HPV epidemiology.

In this context, we assessed the prevalence of alpha, beta, and gamma HPVs in oral rinses from HIV-infected and uninfected MSM using HPV genotyping assays. HPV diversity was further characterized in a subset of HIV-infected and uninfected MSM using a next-generation sequencing (NGS)-based method.

## 2. Materials and Methods

### 2.1. Study Population

From November 2014 to February 2018, MSM attendees of the Sexually Transmitted Infections (STI)/HIV Unit of the San Gallicano Dermatological Institute IRCCS (Rome, Italy) were enrolled to participate in the Oral/Oropharyngeal HPV in Men At Risk (OHMAR) study. The enrollment criteria for this study are described elsewhere [21]. Information regarding sociodemographic and sexual behaviors was collected through face-to-face interviews. HIV-related data were retrieved from medical records.

Written informed consent was obtained from the participants. The study was cleared by the institutional Ethics Committee, I.F.O. Section-Fondazione Bietti (CE/417/14, 13-05-2014). All procedures were performed in accordance with the Declaration of Helsinki.

### 2.2. Oral Sample Collection

Oral rinse-and-gargles were collected as described previously [21]. Briefly, participants rinsed and gargled with 15 mL of Listerine^®^ (McNeil Consumer Healthcare division of Johnson & Johnson, Pomezia, Italy) for 30 s. Samples were centrifuged and washed twice with PreservCyt solution (Hologic, Pomezia, Italy) to remove the mouthwash. The cell pellet was resuspended in 2 mL of PreservCyt. Aliquots were stored at −80 °C for HPV-DNA testing.

### 2.3. Alpha, Beta, and Gamma HPV Detection and Genotyping

The Linear Array HPV Genotyping Test (Roche Molecular Diagnostics, Milan, Italy) was used to detect alpha HPVs (37 types), namely HPV6, 11, 16 18, 26, 31, 33, 35, 39, 40, 42, 45, 51, 52, 53, 54, 55, 56, 58, 59, 61, 62, 64, 66, 67, 68, 69, 70, 71, 72, 73, 81, 82, 83, 84, IS39, and CP6108, following the manufacturer’s instructions. Viral DNA corresponding to 46 beta HPVs and 52 gamma HPVs (listed in Appendix A) were detected using type-specific multiplex genotyping (TS-MPG) assays (IARC, Lyon, France), as previously described [23]. Amplification of beta-globin was used as a control of template quality both for the Linear Array and the TS-MPG assays.

### 2.4. Next-Generation Sequencing for PV Characterization

DNA extracted from oral rinses of the first 30 consecutive HIV-positive and 30 consecutive HIV-negative MSM was amplified using three different sets of primers, namely, the original consensus primer pair FAP59/64 (FAP) [24], the CUT primers [25], and a new set of degenerated FAP primers (FAPM1 primer mix) [26]. The resulting amplicons, ranging from 370 bp (CUT) to 480 bp (FAP and FAPM1), were visualized by electrophoresis on a 2% agarose gel and purified using the QIAquick gel extraction kit according to the manufacturer’s instructions (QIAGEN, Hilden, Germany). Purified PCR amplicons were divided into six different pools (Table 1). After an additional purification step using the Agencourt AMPure XP PCR purification kit (Beckman Coulter, Brea, CA, USA), six NGS libraries were prepared using the Nextera^TM^ DNA Flex Library preparation kit (Illumina, San Diego, CA, USA). Illumina MiSeq dual-indexed adapters (Illumina, San Diego, CA, USA) were added to each PCR pool. The library sizes were checked using the Bioanalyzer 2100 Expert (Agilent, Santa Clara, CA, USA) with a high sensitivity DNA assay. NGS analysis was performed on 4 nM of our DNA pooled library using an Illumina MiSeq instrument (2X150 paired-end reads with the Illumina MiSeq kit v3). To enrich the diversity of the libraries, 10% of PhiX (Illumina, San Diego, CA, US) was added to the NGS reaction.

### 2.5. Bioinformatic Analysis of NGS Data

Bioinformatic analysis was performed as previously described [26,27]. Details of the bioinformatic pipeline named “PVAmpliconFinder” and the parameters used can be found at *https://github.com/IARCbioinfo/PVAmpliconFinder*. All the results in this study were based on the identification of the sequences following the homology-based classification using the evolutionary placement algorithm (EPA) in Randomized Axelerated Maximum Likelihood (RAxML) [28,29], hereafter referred to as RAxML-EPA.

The initial tree used to infer the classification of the PV sequence was built based on 517 L1 sequences available in the PaVe database [30]. Only the longest sequence was considered for RAxML-EPA classification, where several singlets or contigs were clustered together by a PVAmpliconFinder.

### 2.6. Statistical Analysis

Descriptive statistics were computed for the variables of interest to provide summarized descriptions of the study groups. To assess prevalence of infection, individuals were considered to be positive for alpha, beta, or gamma HPVs when they harbored at least one genotype of the respective genus. The different HPVs were assigned to the respective species according to the International Human Papillomavirus Reference Center [31]. To test the statistical significance of differences between HIV-infected and HIV-uninfected MSM regarding HPV prevalence (overall, species-specific and type-specific), a chi-square test was used. A *p* value < 0.05 was set as a threshold for statistical significance. Analyses were performed using MedCalc Statistical Software version 19.3.1 (MedCalc Software Ltd., Ostend, Belgium; https://www.medcalc.org; 2020).

## 3. Results

### 3.1. Study Population

A total of 310 MSM were recruited. Of these, 117 were HIV-infected subjects (37.7%), for the most part on cART (110, 94.0%), with a median number of nadir and a current CD4+ T-cell count of 300 (IQR: 202–403) and 636 cells/mm3 (IQR: 473–812), respectively. Sociodemographic and behavioral characteristics of the participants in the OHMAR study were detailed in a previous report [32]. To summarize, HIV-infected subjects were significantly older (median age: 43 vs. 39 years, *p* = 0.005) and reported a significantly lower number of recent oral sex partners (median of two vs. four, *p* = 0.003) than the HIV-uninfected counterparts. HIV-uninfected MSM reported more frequently to perform receptive oral sex (93.2% vs. 87.2%, *p* = 0.046) and oral sex with occasional partners compared to their HIV-infected counterparts (85.6% vs. 73.5%, *p* = 0.01).

### 3.2. Prevalence of Oral Infection by Alpha, Beta, and Gamma HPVs

**Figure 1:**
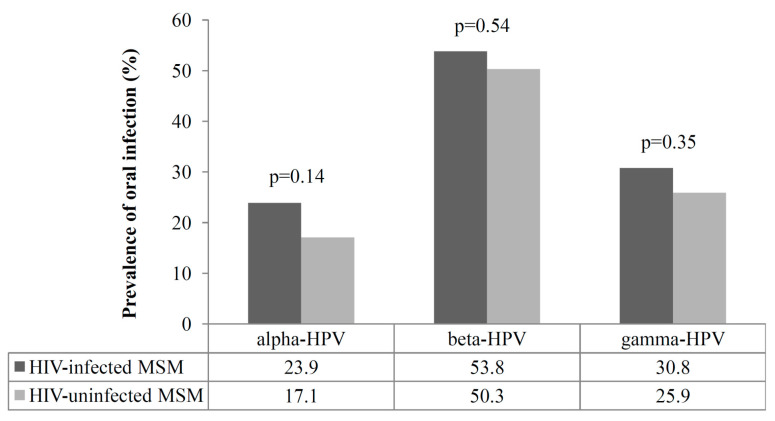
Prevalence of alpha, beta, and gamma HPVs in the oral samples collected from 310 men who have sex with men (MSM), according to HIV status; *p* values for the comparison between HIV-infected and uninfected subjects are shown.

All of the oral rinses gave a valid HPV test result. Prevalence of oral infection by each genus according to HIV status is shown in Figure 1. Overall, 28/117 (23.9%) HIV-infected and 33/193 (17.1%) uninfected MSM were positive for at least one alpha type, with no significant difference between the two groups (*p* = 0.14). The respective figures for beta HPVs were 63/117 (53.8%), and 97/193 (50.3%), *p* = 0.54. Gamma HPVs were detected in 36/117 (30.8%) HIV-infected and 50/193 (25.9%) HIV-uninfected MSM (*p* = 0.35). Beta HPVs were significantly more frequent than the alpha types both among HIV-infected (53.8% vs. 23.9%, *p* < 0.0001) and uninfected subjects (50.3% vs. 17.1%, *p* < 0.0001).

The frequency of multiple infections (>1 type of the same genus) did not differ significantly according to the HIV status for any of the three genera. Multiple infections were more frequent in beta (52.4% and 50.5% of the beta-positive HIV-infected and uninfected MSM, respectively; *p* = 0.48), than alpha (35.7% and 27.3% of the alpha-positive HIV-infected and uninfected MSM, respectively; *p* = 0.82) and gamma HPV infections (36.1% and 38.1% of the gamma-positive HIV-infected and uninfected MSM, respectively; *p* = 0.86). No differences were observed between HIV-infected and uninfected MSM regarding the median number of genotypes found in the multiple infections (three vs. three for alpha, three vs. four for beta, and two vs. two for gamma HPVs, respectively).

Species-specific and type-specific prevalence are shown in Table 2 for alpha, Table 3 for beta and Table 4 for gamma HPVs. Alpha-3 (10.2%) and alpha-9 (6.2%) represented the most frequently detected alpha species in HIV-infected and uninfected MSM, respectively (Table 2). None of the alpha species were significantly different between the two study groups. HPV55, 72, 84, and IS39 were the most common alpha types among HIV-infected individuals (3.4% each), whereas HPV16 was the most frequent type in the HIV-uninfected counterparts (4.1%).

Beta-1 (39.3%) and beta-2 (35.2%) represented the most frequent beta species in HIV-infected and uninfected MSM, respectively (Table 3). None of the beta species were significantly different between the two study groups. HPV5 was the most common beta type in both groups.

Gamma-10 was the most frequent gamma species in both HIV-infected (11.1%) and uninfected MSM (8.8%) (Table 4). A significant difference was observed in the prevalence of gamma-1 and gamma-2 species, which were enriched among HIV-infected individuals. The most frequent gamma types were HPV130 and 133 in HIV-infected MSM (5.1% each), whereas HPV133 and 175 represented the predominant types in their HIV-uninfected counterparts (4.7% each).

### 3.3. NGS Data and PV Sequence Analyses 

To comprehensively characterize the spectrum of HPV types present in the oral cavity, an NGS-based approach was applied to a subset of samples. The oral specimens from 30 HIV-positive and 30 HIV-negative patients were processed for PCR amplification using three different primer sets all targeting L1 Open Reading Frame (ORF). Pooled PCR amplicons were sequenced (Table 1), generating a total of 2,620,725 reads identified as related to PV sequences (Table 5). Data analysis evidenced that these reads comprised 2,620,537 reads (99.99%) from known PVs, while the remaining reads corresponded to novel putative PVs (<90% identity with L1 ORF of any known PV). A total of 961,331 reads (36.7%) corresponded to alpha-PVs, followed by gamma-PVs (880,964 reads, 33.6%), beta-PVs (777,200 reads, 29.6%), and unclassified PVs (1230 reads, 0.047%). In summary, the 2,620,725 reads were representative of 8 unknown and 146 known PVs (145 officially recognized, namely 16 alpha-PVs, 53 beta-PVs, 76 gamma-PVs, and one remaining unclassified) (Table 5 and Table S1).

The distribution of all known HPV types detected in HIV-positive and HIV-negative patients is shown in Table 5 and Table S1. Regarding alpha HPVs, a higher cumulative number of reads (*n* = 932,336) was generated by all three PCR protocols in pools consisting of oral samples from HIV-infected individuals compared to the HIV-uninfected ones (*n* = 28,995). Reads were representative of eight different alpha species, of which alpha-1 reads (*n* = 566,106) representative of HPV32 were the most abundant in the HIV-infected group, while alpha-9 reads (*n* = 28,909), mostly from HPV16 (*n* = 28,474), were predominant in the HIV-uninfected group (Table 5 and Table S1).

Conversely, the cumulative number of reads representative of beta-PV sequences were more abundant in pools of HIV-negative specimens (*n* = 689,080) compared to HIV-positive ones (*n* = 87,932) (Table 5 and Table S1). Reads from all six beta species (beta-1 to beta-6) in addition to 11 unreferenced beta HPVs were identified; they were representative of 52 HPVs and one animal PV (species beta-6). Reads from beta-1 (i.e., HPV21, HPV24, and HPV105) and beta-2 (i.e., HPV9, HPV23, and HPV111) species were abundant in both MSM groups (Table S1).

The cumulative number of reads representative of gamma-PV types were more abundant in pools from the HIV-uninfected group (*n* = 803,955) compared to the HIV-infected group (*n* = 77,009). Reads from 12 of 27 known gamma species, which were representative of 25 HPVs, were identified in addition to 50 unreferenced gamma HPVs. Reads from gamma-10 HPV130 (*n* = 18,883), HPV133 (*n* = 7868), and HPV180 (*n* = 21,565) were predominant in the HIV-positive group and were 80 times more abundant compared to the HIV-negative group (Table 5 and Table S1). Altogether, a similar number of PVs were found in both HIV-positive and HIV-negative groups with 12 vs. 10 alpha-PVs, 38 vs. 46 beta-PVs, and 56 vs. 45 gamma-PVs, respectively (Table 5).

Among the unknown PV sequences, six putative novel sequences were found in NGS pools from HIV-negative specimens (75.0%), while two others were found in HIV-positive (25.0%) specimens (Table 5 and Table S2). Putative new HPV types in HIV-negative samples were related to HPV 23, 38, 110, and 120 (all beta-2 species), and unreferenced HPV-mSK111 and HPV-mm292c88. In HIV-positive patients, the new PV sequences were related to HPV98 (beta-1) and HPV122 (beta-2).

## 4. Discussion

This study investigated the presence of a wide spectrum of mucosal and cutaneous HPVs in oral samples of HIV-infected and uninfected MSM. Results obtained with specific HPV genotyping assays showed that cutaneous HPVs are more prevalent than mucosal types in the oral cavity. In both study groups, beta HPVs were the most abundant types, being significantly more frequent than alpha HPVs. Independently of the HIV status, HPV5 was the most frequent beta type. Beta HPV8 and HPV21 were significantly more prevalent in HIV-uninfected subjects. High oral prevalence of both HPV5 and HPV8 were reported elsewhere [33].

Beta HPV prevalence in our participants exceeded that observed in other studies in male [6,34] and female populations [7], and both beta and gamma HPVs were much more frequent in our study groups than in men of a population-based study of asymptomatic individuals [5]. Demographic characteristics, predominantly age, sex, and ethnicity, may contribute to explain these differences. The higher prevalence of cutaneous HPVs in MSM might be also due to a higher risk of acquiring the infection or a longer persistence of the infection in these individuals. Longitudinal studies are needed to cast light on these aspects. Notably, beta HPVs were nearly twice as common in the oral cavity than in the anal canal of HIV-infected and uninfected MSM [3], suggesting a different natural history of beta HPV infection at these anatomic sites or a role for different sexual exposures at oral and anal sites. Evaluation of concordance of infection across multiple sites may help clarify these points.

Despite the fact that HIV-infected individuals usually show higher incidence, prevalence, and persistence of oral and anogenital HPV infections by mucosal types [2,3,20,33,35], we observed no effect of HIV status on the oral prevalence of alpha HPVs. We also observed no significant difference between HIV-infected and uninfected subjects regarding the prevalence of beta and gamma types. Similarly, we previously found that anal prevalence of cutaneous HPVs in MSM did not change significantly with HIV status [3]. Regarding individual genotypes, only alpha HPV72 and IS39 and gamma HPV200 were significantly more represented in HIV-infected MSM. However, it must be noted that, in this study, HIV-infected participants were, for the most part, on successful cART regimens, and they thus were immunorestored and aviremic. Lack of HIV-infected individuals with severe immunosuppression may be the reason why no significant differences were observed when considering HIV status.

We also used an NGS-based broad-spectrum strategy [26] to detect known and new HPV types in a subset of HIV-positive and negative samples. Regarding known HPVs, the NGS data confirmed that all three genera were highly abundant in both MSM groups with a total of 16 alpha, 52 beta, and 75 gamma HPVs identified, meaning that approximately three quarters of beta HPVs and a quarter of gamma HPVs listed in the Papilloma Virus Episteme (PaVE) were detected in the oral cavity (https://pave.niaid.nih.gov/, on 1 April 2020). These data suggest that a wide spectrum of cutaneous HPV types inhabits the oral mucosa, as reported elsewhere [5,33,36], indicating that beta and gamma HPVs may not have a strict tropism for skin epithelia, but may also be able to infect mucosal epithelia, as discussed in [34,37] and reviewed in [38].

Although similar numbers of alpha-, beta-, and gamma-PV types were found in the oral cavity of both groups, reads from alpha-PVs were over-represented in pools from HIV-positive patients, while reads from beta- and gamma-PVs were more abundant in HIV-negative individuals. The latter group reported they had oral sex more frequently than their HIV-infected counterparts, thus suggesting a preferential sexual transmission route for beta and gamma HPVs. Some HPVs from beta-1 and beta-2 species (e.g., HPV21 and HPV23) were abundantly represented in both MSM groups, suggesting a stronger ability of specific types to evade immune surveillance and colonize the oral mucosa, as shown for high-risk HPV types [39]. Additionally, the NGS data revealed that HPV types from gamma-10 species were highly represented in the HIV-infected group, with HPV130 and HPV133 being the most abundant, thereby confirming our prevalence data. These findings suggest that some specific gamma types are frequently found in the oral cavity and thus deserve further investigation to establish whether this may suggest a possible tropism for this site.

NGS also led to the identification of eight new putative HPVs, all belonging to the beta genus. Our previous study performed on skin and oral gargles from healthy individuals, using the same methodology, revealed the presence of 105 putative novel PV types, among which eight beta and two gamma HPVs were found in the oral cavity [26]. Other studies evidenced the presence of additional new beta and gamma HPVs at this site [5,33], thus increasing our knowledge of HPV diversity in the oral mucosa. Importantly, among the large number of beta and gamma HPVs characterized in recent years, some may display transforming activity, as shown in vivo and in vitro for HPVs belonging to beta-3 species [40,41].

This study has some limitations. Firstly, HIV-infected patients were mainly immunorestored by cART, thus we were unable to reveal potential effects of HIV-related immunosuppression on oral HPV infection. Secondly, NGS analysis was only performed on a subset of the study samples. Finally, with the NGS pool-based strategy, it is not possible to determine the PV diversity within an individual, nor determine prevalence.

## 5. Conclusions

In conclusion, our study evidenced that a wide spectrum of HPV types can be detected in oral samples of MSM and that beta HPVs represent the most abundant types, exceeding the prevalence of both alpha and gamma HPVs. Our findings confirm that cutaneous HPVs are frequently observed in mucosal epithelia. A robust NGS strategy for PV analysis confirmed the high diversity of HPVs in oral infection, with the alpha genus being the least diverse compared to the cutaneous genera. Through this strategy, a number of putative novel PVs were also identified.

Further studies are necessary to clarify the natural history and transmission modalities of cutaneous HPVs in the oral cavity and, more importantly, to investigate their possible pathogenic role at this site.

## Figures and Tables

**Table 1:** Description of the next-generation sequencing (NGS) pools. Human Papillomavirus (HPV)-positive PCR products were grouped into six NGS pools according to HIV status and applied PCR protocol.

NGS Pool	PCR Protocol	Specimen HIV Status	Total Number *
1	CUT	HIV−	29
2	CUT	HIV+	30
3	FAP	HIV−	30
4	FAP	HIV+	28
5	FAPM1	HIV−	18
6	FAPM1	HIV+	14

* Positive PCR products.

**Table 2:** Species-specific and type-specific prevalence of alpha HPVs in single and multiple oral infections among the 310 MSM of the Oral/Oropharyngeal HPV in Men At Risk (OHMAR) study according to HIV status.

Alpha Species/Type	HIV-Infected *n* = 117 *n* (%)			HIV-Uninfected *n* = 193 *n* (%)			
	Single Infection	Multiple Infection	Total	Single Infection	Multiple Infection	Total	*p* Value ^a^
**Any alpha-3**	**6 (5.1)**	**6 (5.1)**	**12 (10.2)**	**6 (3.1)**	**4 (2.1)**	**10 (5.2)**	
61	1 (0.9)	1 (0.9)	2 (1.7)	0 (0.0)	0 (0.0)	0 (0.0)	
62	2 (1.7)	0 (0.0)	2 (1.7)	1 (0.5)	0 (0.0)	1 (0.5)	
72	2 (1.7)	2 (1.7)	4 (3.4)	0 (0.0)	1 (0.5)	1 (0.5)	0.049
81	0 (0.0)	1 (0.9)	1 (0.9)	0 (0.0)	0 (0.0)	0 (0.0)	
83	0 (0.0)	0 (0.0)	0 (0.0)	1 (0.5)	1 (0.5)	2 (1.0)	
84	1 (0.9)	3 (2.6)	4 (3.4)	0 (0.0)	2 (1.0)	2 (1.0)	
89 (CP6108)	0 (0.0)	3 (2.6)	3 (2.6)	4 (2.1)	1 (0.5)	5 (2.6)	
**Any alpha-5**	**2 (1.7)**	**2 (1.7)**	**4 (3.4)**	**1 (0.5)**	**1 (0.5)**	**2 (1.0)**	
26	0 (0.0)	0 (0.0)	0 (0.0)	1 (0.5)	0 (0.0)	1 (0.5)	
51	0 (0.0)	2 (1.7)	2 (1.7)	0 (0.0)	0 (0.0)	0 (0.0)	
69	1 (0.9)	0 (0.0)	1 (0.9)	0 (0.0)	0 (0.0)	0 (0.0)	
82	1 (0.9)	1 (0.9)	2 (1.7)	0 (0.0)	1 (0.5)	1 (0.5)	
**Any alpha-6**	**0 (0.0)**	**3 (2.6)**	**3 (2.6)**	**6 (3.1)**	**4 (2.1)**	**10 (5.2)**	
53	0 (0.0)	1 (0.9)	1 (0.9)	1 (0.5)	2 (1.0)	3 (1.5)	
56	0 (0.0)	0 (0.0)	0 (0.0)	2 (1.0)	2 (1.0)	4 (2.1)	
66	0 (0.0)	2 (1.7)	2 (1.7)	3 (1.5)	0 (0.0)	3 (1.5)	
**Any alpha-7**	**5 (4.3)**	**4 (3.4)**	**9 (7.7)**	**2 (1.0)**	**6 (3.1)**	**8 (4.1)**	
18	1 (0.9)	2 (1.7)	3 (2.6)	1 (0.5)	0 (0.0)	1 (0.5)	
39	1 (0.9)	1 (0.9)	2 (1.7)	0 (0.0)	0 (0.0)	0 (0.0)	
45	0 (0.0)	1 (0.9)	1 (0.9)	0 (0.0)	3 (1.5)	3 (1.5)	
59	0 (0.0)	2 (1.7)	2 (1.7)	1 (0.5)	0 (0.0)	1 (0.5)	
68	2 (1.7)	0 (0.0)	2 (1.7)	0 (0.0)	2 (1.0)	2 (1.0)	
70	1 (0.9)	0 (0.0)	1 (0.9)	0 (0.0)	1 (0.5)	1 (0.5)	
**Any alpha-9**	**0 (0.0)**	**3 (2.6)**	**3 (2.6)**	**6 (3.1)**	**6 (3.1)**	**12 (6.2)**	
16	0 (0.0)	3 (2.6)	3 (2.6)	2 (1.0)	6 (3.1)	8 (4.1)	
33	0 (0.0)	1 (0.9)	1 (0.9)	2 (1.0)	0 (0.0)	2 (1.0)	
35	0 (0.0)	0 (0.0)	0 (0.0)	1 (0.5)	0 (0.0)	1 (0.5)	
58	0 (0.0)	0 (0.0)	0 (0.0)	1 (0.5)	0 (0.0)	1 (0.5)	
**Any alpha-10**	**0 (0.0)**	**3 (2.6)**	**3 (2.6)**	**2 (1.0)**	**0 (0.0)**	**2 (1.0)**	
6	0 (0.0)	1 (0.9)	1 (0.9)	1 (0.5)	0 (0.0)	1 (0.5)	
11	0 (0.0)	2 (1.7)	2 (1.7)	1 (0.5)	0 (0.0)	1 (0.5)	
**Any alpha-11**	**0 (0.0)**	**1 (0.9)**	**1 (0.9)**	**1 (0.5)**	**0 (0.0)**	**1 (0.5)**	
73	0 (0.0)	1 (0.9)	1 (0.9)	1 (0.5)	0 (0.0)	1 (0.5)	
**Any alpha-13**	**0 (0.0)**	**0 (0.0)**	**0 (0.0)**	**0 (0.0)**	**1 (0.5)**	**1 (0.5)**	
54	0 (0.0)	0 (0.0)	0 (0.0)	0 (0.0)	1 (0.5)	1 (0.5)	
**Any alpha-14**	**0 (0.0)**	**1 (0.9)**	**1 (0.9)**	**0 (0.0)**	**0 (0.0)**	**0 (0.0)**	
71	0 (0.0)	1 (0.9)	1 (0.9)	0 (0.0)	0 (0.0)	0 (0.0)	
**Other**	**5 (4.3)**	**3 (2.6)**	**8 (6.8)**	**0 (0.0)**	**2 (1.0)**	**2 (1.0)**	
55 (44 subtype)	3 (2.6)	1 (0.9)	4 (3.4)	0 (0.0)	2 (1.0)	2 (1.0)	
IS39 (82 subtype)	2 (1.7)	2 (1.7)	4 (3.4)	0 (0.0)	0 (0.0)	0 (0.0)	0.01

^a^ for the comparison between the prevalence of the individual species (or types) in HIV-infected and that in HIV-uninfected MSM; Species and individual genotypes not detected in any of the HIV-infected and uninfected MSM are not shown; *p* values are shown only for significant differences; when the prevalence of the species (or individual types) did not differ significantly between HIV-infected and uninfected MSM (*p* > 0.05), the *p* value is not shown; The number (and percentages) of subjects positive for each species may not be the sum of the subjects (and percentages) positive for the individual genotypes included in that species because of multiple infections; Due to rounding, some totals may not correspond with the sum of the separate figures.

**Table 3:** Species-specific and type-specific prevalence of beta HPVs in single and multiple oral infections among the 310 MSM of the OHMAR study according to HIV status.

Beta Species/Type	HIV-Infected *n* = 117 *n* (%)			HIV-Uninfected *n* = 193 *n* (%)			
	Single Infection	Multiple Infection	Total	Single Infection	Multiple Infection	Total	*p* Value ^a^
**Any beta-1**	**17 (14.5)**	**29 (24.8)**	**46 (39.3)**	**21 (10.9)**	**45 (23.3)**	**66 (34.2)**	
5	5 (4.3)	8 (6.8)	13 (11.1)	5 (2.6)	19 (9.8)	24 (12.4)	
8	1 (0.9)	1 (0.9)	2 (1.7)	4 (2.1)	12 (6.2)	16 (8.3)	0.01
12	3 (2.6)	4 (3.4)	7 (6.0)	1 (0.5)	11 (5.7)	12 (6.2)	
14	2 (1.7)	1 (0.9)	3 (2.6)	0 (0.0)	3 (1.5)	3 (1.5)	
19	0 (0.0)	4 (3.4)	4 (3.4)	2 (1.0)	11 (5.7)	13 (6.7)	
20	0 (0.0)	0 (0.0)	0 (0.0)	0 (0.0)	1 (0.5)	1 (0.5)	
21	0 (0.0)	3 (2.6)	3 (2.6)	2 (1.0)	14 (7.3)	16 (8.3)	0.04
24	0 (0.0)	6 (5.1)	6 (5.1)	2 (1.0)	10 (5.2)	12 (6.2)	
25	1 (0.9)	3 (2.6)	4 (3.4)	0 (0.0)	2 (1.0)	2 (1.0)	
36	1 (0.9)	1 (0.9)	2 (1.7)	2 (1.0)	3 (1.5)	5 (2.6)	
47	0 (0.0)	9 (7.7)	9 (7.7)	1 (0.5)	9 (4.7)	10 (5.2)	
98	0 (0.0)	1 (0.9)	1 (0.9)	0 (0.0)	6 (3.1)	6 (3.1)	
99	0 (0.0)	3 (2.6)	3 (2.6)	0 (0.0)	4 (2.1)	4 (2.1)	
105	2 (1.7)	7 (6.0)	9 (7.7)	2 (1.0)	12 (6.2)	14 (7.3)	
124	2 (1.7)	5 (4.3)	7 (6.0)	0 (0.0)	6 (3.1)	6 (3.1)	
143	0 (0.0)	1 (0.9)	1 (0.9)	0 (0.0)	1 (0.5)	1 (0.5)	
**Any beta-2**	**11 (9.4)**	**27 (23.1)**	**38 (32.5)**	**24 (12.4)**	**44 (22.8)**	**68 (35.2)**	
9	3 (2.6)	5 (4.3)	8 (6.8)	0 (0.0)	8 (4.1)	8 (4.1)	
15	0 (0.0)	3 (2.6)	3 (2.6)	1 (0.5)	4 (2.1)	5 (2.6)	
17	0 (0.0)	3 (2.6)	3 (2.6)	0 (0.0)	5 (2.6)	5 (2.6)	
22	0 (0.0)	1 (0.9)	1 (0.9)	0 (0.0)	3 (1.5)	3 (1.5)	
23	0 (0.0)	1 (0.9)	1 (0.9)	0 (0.0)	3 (1.5)	3 (1.5)	
38	2 (1.7)	8 (6.8)	10 (8.5)	7 (3.6)	13 (6.7)	20 (10.4)	
80	0 (0.0)	0 (0.0)	0 (0.0)	0 (0.0)	1 (0.5)	1 (0.5)	
100	0 (0.0)	3 (2.6)	3 (2.6)	2 (1.0)	10 (5.2)	12 (6.2)	
107	0 (0.0)	0 (0.0)	0 (0.0)	0 (0.0)	1 (0.5)	1 (0.5)	
110	0 (0.0)	6 (5.1)	6 (5.1)	2 (1.0)	10 (5.2)	12 (6.2)	
111	0 (0.0)	4 (3.4)	4 (3.4)	1 (0.5)	10 (5.2)	11 (5.7)	
120	1 (0.9)	5 (4.3)	6 (5.1)	2 (1.0)	4 (2.1)	6 (3.1)	
122	1 (0.9)	4 (3.4)	5 (4.3)	3 (1.5)	14 (7.3)	17 (8.8)	
145	1 (0.9)	3 (2.6)	4 (3.4)	0 (0.0)	7 (3.6)	7 (3.6)	
151	1 (0.9)	2 (1.7)	3 (2.6)	1 (0.5)	8 (4.1)	9 (4.7)	
159	1 (0.9)	1 (0.9)	2 (1.7)	0 (0.0)	3 (1.5)	3 (1.5)	
174	1 (0.9)	4 (3.4)	5 (4.3)	5 (2.6)	6 (3.1)	11 (5.7)	
**Any beta-3**	**2 (1.7)**	**10 (8.5)**	**12 (10.3)**	**3 (1.5)**	**12 (6.2)**	**15 (7.7)**	
49	0 (0.0)	3 (2.6)	3 (2.6)	0 (0.0)	4 (2.1)	4 (2.1)	
75	0 (0.0)	2 (1.7)	2 (1.7)	1 (0.5)	3 (1.5)	4 (2.1)	
76	1 (0.9)	6 (5.1)	7 (6.0)	2 (1.0)	6 (3.1)	8 (4.1)	
115	1 (0.9)	0 (0.0)	1 (0.9)	0 (0.0)	0 (0.0)	0 (0.0)	
**Any beta-5**	**0 (0.0)**	**0 (0.0)**	**0 (0.0)**	**0 (0.0)**	**2 (1.0)**	**2 (1.0)**	
96	0 (0.0)	0 (0.0)	0 (0.0)	0 (0.0)	2 (1.0)	2 (1.0)	

^a^ for the comparison between the prevalence of the individual species (or types) in HIV-infected and that in HIV-uninfected MSM; Species and individual genotypes not detected in any of the HIV-infected and uninfected MSM are not shown; *p* values are shown only for significant differences; when the prevalence of the species (or individual types) did not differ significantly between HIV-infected and uninfected MSM (*p* > 0.05), the *p* value is not shown; The number (and percentages) of subjects positive for each species may not be the sum of the subjects (and percentages) positive for the individual genotypes included in that species because of multiple infections; Due to rounding, some totals may not correspond with the sum of the separate figures.

**Table 4:** Species-specific and type-specific prevalence of gamma HPVs in single and multiple oral infections among the 310 MSM of the OHMAR study according to HIV status.

Gamma Species/Type	HIV-Infected *n* = 117 *n* (%)			HIV-Uninfected *n* = 193 *n* (%)			
	Single Infection	Multiple Infection	Total	Single Infection	Multiple Infection	Total	*p* Value ^a^
**Any gamma-1**	**2 (1.7)**	**2 (1.7)**	**4 (3.4)**	**0 (0.0)**	**1 (0.5)**	**1 (0.5)**	0.049
4	1 (0.9)	1 (0.9)	2 (1.7)	0 (0.0)	1 (0.5)	1 (0.5)	
65	1 (0.9)	1 (0.9)	2 (1.7)	0 (0.0)	0 (0.0)	0 (0.0)	
**Any gamma-2**	**4 (3.4)**	**1 (0.9)**	**5 (4.3)**	**1 (0.5)**	**0 (0.0)**	**1 (0.5)**	0.02
200	4 (3.4)	1 (0.9)	5 (4.3)	1 (0.5)	0 (0.0)	1 (0.5)	0.02
**Any gamma-6**	**2 (1.7)**	**0 (0.0)**	**2 (1.7)**	**0 (0.0)**	**1 (0.5)**	**1 (0.5)**	
108	2 (1.7)	0 (0.0)	2 (1.7)	0 (0.0)	1 (0.5)	1 (0.5)	
**Any gamma-7**	**1 (0.9)**	**3 (2.6)**	**4 (3.4)**	**2 (1.0)**	**7 (3.6)**	**9 (4.7)**	
123	0 (0.0)	1 (0.9)	1 (0.9)	0 (0.0)	1 (0.5)	1 (0.5)	
134	0 (0.0)	1 (0.9)	1 (0.9)	1 (0.5)	0 (0.0)	1 (0.5)	
149	1 (0.9)	2 (1.7)	3 (2.6)	0 (0.0)	5 (2.6)	5 (2.6)	
170	0 (0.0)	0 (0.0)	0 (0.0)	1 (0.5)	1 (0.5)	2 (1.0)	
**Any gamma-8**	**0 (0.0)**	**1 (0.9)**	**1 (0.9)**	**0 (0.0)**	**3 (1.5)**	**3 (1.5)**	
112	0 (0.0)	0 (0.0)	0 (0.0)	0 (0.0)	1 (0.5)	1 (0.5)	
164	0 (0.0)	0 (0.0)	0 (0.0)	0 (0.0)	1 (0.5)	1 (0.5)	
168	0 (0.0)	1 (0.9)	1 (0.9)	0 (0.0)	1 (0.5)	1 (0.5)	
**Any gamma-10**	**5 (4.3)**	**8 (6.8)**	**13 (11.1)**	**5 (2.6)**	**12 (6.2)**	**17 (8.8)**	
121	0 (0.0)	1 (0.9)	1 (0.9)	1 (0.5)	4 (2.1)	5 (2.6)	
130	3 (2.6)	3 (2.6)	6 (5.1)	1 (0.5)	2 (1.0)	3 (1.5)	
133	2 (1.7)	4 (3.4)	6 (5.1)	2 (1.0)	7 (3.6)	9 (4.7)	
180	0 (0.0)	2 (1.7)	2 (1.7)	1 (0.5)	7 (3.6)	8 (4.1)	
**Any gamma-11**	**0 (0.0)**	**2 (1.7)**	**2 (1.7)**	**1 (0.5)**	**0 (0.0)**	**1 (0.5)**	
169	0 (0.0)	2 (1.7)	2 (1.7)	1 (0.5)	0 (0.0)	1 (0.5)	
**Any gamma-12**	**1 (0.9)**	**2 (1.7)**	**3 (2.6)**	**4 (2.1)**	**7 (3.6)**	**11 (5.7)**	
127	0 (0.0)	0 (0.0)	0 (0.0)	1 (0.5)	0 (0.0)	1 (0.5)	
132	1 (0.9)	2 (1.7)	3 (2.6)	1 (0.5)	6 (3.1)	7 (3.6)	
148	0 (0.0)	1 (0.9)	1 (0.9)	2 (1.0)	1 (0.5)	3 (1.5)	
**Any gamma-13**	**0 (0.0)**	**2 (1.7)**	**2 (1.7)**	**4 (2.1)**	**3 (1.5)**	**7 (3.6)**	
128	0 (0.0)	2 (1.7)	2 (1.7)	4 (2.1)	3 (1.5)	7 (3.6)	
**Any gamma-15**	**1 (0.9)**	**2 (1.7)**	**3 (2.6)**	**1 (0.5)**	**4 (2.1)**	**5 (2.6)**	
179	1 (0.9)	2 (1.7)	3 (2.6)	1 (0.5)	4 (2.1)	5 (2.6)	
**Any gamma-18**	**1 (0.9)**	**3 (2.6)**	**4 (3.4)**	**2 (1.0)**	**2 (1.0)**	**4 (2.1)**	
156	1 (0.9)	3 (2.6)	4 (3.4)	2 (1.0)	2 (1.0)	4 (2.1)	
**Any gamma-19**	**1 (0.9)**	**0 (0.0)**	**1 (0.9)**	**0 (0.0)**	**1 (0.5)**	**1 (0.5)**	
161	0 (0.0)	0 (0.0)	0 (0.0)	0 (0.0)	1 (0.5)	1 (0.5)	
162	1 (0.9)	0 (0.0)	1 (0.9)	0 (0.0)	0 (0.0)	0 (0.0)	
**Any gamma-22**	**3 (2.6)**	**1 (0.9)**	**4 (3.4)**	**1 (0.5)**	**2 (1.0)**	**3 (1.5)**	
172	3 (2.6)	1 (0.9)	4 (3.4)	1 (0.5)	2 (1.0)	3 (1.5)	
**Any gamma-23**	**1 (0.9)**	**1 (0.9)**	**2 (1.7)**	**4 (2.1)**	**5 (2.6)**	**9 (4.7)**	
175	1 (0.9)	1 (0.9)	2 (1.7)	4 (2.1)	5 (2.6)	9 (4.7)	
**Any gamma-25**	**1 (0.9)**	**3 (2.6)**	**4 (3.4)**	**3 (1.5)**	**1 (0.5)**	**4 (2.1)**	
184	1 (0.9)	3 (2.6)	4 (3.4)	3 (1.5)	1 (0.5)	4 (2.1)	
**Any gamma-27**	**0 (0.0)**	**2 (1.7)**	**2 (1.7)**	**0 (0.0)**	**2 (1.0)**	**2 (1.0)**	
201	0 (0.0)	2 (1.7)	2 (1.7)	0 (0.0)	2 (1.0)	2 (1.0)	
**Other**	**0 (0.0)**	**3 (2.6)**	**3 (2.6)**	**3 (1.5)**	**2 (1.0)**	**5 (2.6)**	
SD2 ^b^	0 (0.0)	3 (2.6)	3 (2.6)	3 (1.5)	2 (1.0)	5 (2.6)	

^a^ for the comparison between the prevalence of the individual species (or types) in HIV-infected and that in HIV-uninfected MSM; ^b^ no species assigned; Species and individual genotypes not detected in any of the HIV-infected and uninfected MSM are not shown; *p* values are shown only for significant differences; when the prevalence of the species (or individual types) did not differ significantly between HIV-infected and uninfected MSM (*p* > 0.05), the *p* value is not shown; The number (and percentages) of subjects positive for each species may not be the sum of the subjects (and percentages) positive for the individual genotypes included in that species because of multiple infections; Due to rounding, some totals may not correspond with the sum of the separate figures.

**Table 5:** Known and putative novel PV sequences in HIV-infected and HIV-uninfected samples. The number of sequences and corresponding reads are reported for alpha, beta, gamma, and unclassified PVs, stratified according to the primer set, by Randomized Axelerated Maximum Likelihood-evolutionary placement algorithm (RAxML-EPA) taxonomic classification according to HIV status.

	HIV-Infected *n* (reads)				HIV-Uninfected *n* (reads)				
PV Genus	Unique PV	FAP	FAPM1	CUT	Unique PV	FAP	FAPM1	CUT	Total Unique
**alpha**	12 (932,336)	2 (40,556)	4 (860,360)	11 (31,420)	10 (28,995)	1 (4)	4 (16,371)	7 (12,620)	16 (961,331)
**beta**	38 (87,932)	7 (9543)	13 (2416)	35 (75,973)	46 (689,080)	16 (5828)	23 (585,069)	39 (98,183)	53 (777,012)
**gamma**	56 (77,009)	14 (6888)	20 (44,186)	41 (25,939)	45 (803,955)	8 (34,910)	17 (739,439)	35 (29,606)	76 (880,964)
**unclassified**	0 (0)	0 (0)	0 (0)	0 (0)	1 (1230)	0 (0)	0 (0)	1 (1230)	1 (1230)
**Sub-total**	106 (1,097,277)	23 (56,983)	37 (906,962)	87 (133,332)	101 (1,522,260)	25 (40,742)	44 (1,340,879)	82 (141,639)	146 (2,620,537)
**Putative new beta**	2 (9)	0 (0)	1 (2)	1 (7)	6 (179)	2 (43)	4 (125)	1 (11)	8 (188)
**Total**	108 (1,097,286)	23 (56,983)	38 (906,964)	88 (133,339)	107 (1,522,439)	27 (40,785)	48 (1,341,004)	83 (141,650)	154 (2,620,725)

## Supplementary Materials

The following are available online at https://www.mdpi.com/1999-4915/12/8/899/s1: Table S1: Known PV types identified by three different NGS-based protocols in oral rinses of HIV-infected and uninfected MSM; Table S2: Unknown PV types identified by three different NGS-based protocols in oral rinses of HIV-infected and uninfected MSM.

## Appendix A

Viral DNA corresponding to 46 beta HPVs from β-species 1 (5, 8, 12, 14, 19, 20, 21, 24, 25, 36, 47, 93, 98, 99, 105, 118, 124, 143, and 152), β-species 2 (9, 15, 17, 22, 23, 37, 38, 80, 100, 104, 107, 110, 111, 113, 120, 122, 145, 151, 159, and 174), β-species 3 (49, 75, 76, and 115), β-species 4 (92), and β-species 5 (96 and 150), and 52 gamma HPVs from γ-species 1 (4, 65, 95, and 173), γ-species 2 (48 and 200), γ-species 3 (50), γ-species 5 (60, 88), γ-species 6 (101, 103, and 108), γ-species 7 (109, 123, 134, 149, and 170), γ-species 8 (112, 119, 164, and 168), γ-species 9 (116 and 129), γ-species 10 (121, 130, 133, and 180), γ-species 11 (126, 169, 171, and 202), γ-species 12 (127, 132, 148, 165, and 199), γ-species 13 (128), γ-species 14 (131), γ-species 15 (179), γ-species 18 (156), γ-species 19 (161, 162 and 166), γ-species 20 (163), γ-species 21 (167), γ-species 22 (172), γ-species 23 (175), γ-species 24 (178 and 197), γ-species 25 (184), γ-species 27 (201), and SD2 (Genbank accession id: KC113191) were detected using type-specific multiplex genotyping (TS-MPG) assays (IARC, Lyon, France).
